# Possible autoimmune hemolytic anemia induced by secukinumab: a case report

**DOI:** 10.11604/pamj.2022.41.41.32191

**Published:** 2022-01-17

**Authors:** Perdana Aditya Rahman, Handono Kalim, Suci Prawitasari, Fajar Maulana Raharjo

**Affiliations:** 1 Rheumatology - Immunology Division, Department of Internal Medicine, Faculty of Medicine, Universitas Brawijaya, Malang, Indonesia,; 2 Rheumatology-Immunology Division, Department of Internal Medicine, Saiful Anwar General Hospital, Malang, Indonesia,; 3 Department of Dermatovenereology, Faculty of Medicine, Universitas Brawijaya, Malang, Indonesia,; 4 Department of Dermatovenereology, Saiful Anwar General Hospital, Malang, Indonesia,; 5 Faculty of Medicine, Universitas Brawijaya, Malang, Indonesia

**Keywords:** Autoimmune hemolytic anemia, psoriasis, psoriatic arthritis, secukinumab, case report

## Abstract

Secukinumab, an anti-IL-17 monoclonal antibody, has been used to treat psoriasis and psoriatic arthritis since 2015. Several adverse events were reported, such as diarrhea, upper respiratory tract infection, middle ear infection, and neutropenia. Here we report a probable case of autoimmune hemolytic anemia in a 39 years old male with psoriasis and psoriatic arthritis treated with secukinumab. Hemolytic anemia detected after first maintenance dose after completion of induction dose of secukinumab. The patient also had other comorbids, soft tissue infection that also predisposed to autoimmune hemolytic anemia, but secukinumab is still a possible etiology for drug-induced autoimmune hemolytic anemia based on Naranjo´s score. The patient decided to continue secukinumab treatment, interestingly hemoglobin levels improved.

## Introduction

In recent years, numerous targeted immunotherapies have been authorized for the treatment of psoriasis, arthritis, and these same immune pathways are being investigated for their potential application in the treatment of psoriatic arthritis. One of the routes identified as a potential therapeutic target for psoriatic arthritis is the interleukin-17A inflammatory cytokine pathway (IL-17A) [1]. The IL-17 signaling pathway is involved in the pathophysiology of psoriasis arthritis caused in the synovial fluid of patients with psoriasis arthritis has an enrichment of IL-17A and IL-17RA [2]. Secukinumab is a human anti-interleukin-17A monoclonal antibody that has been recognized for the treatment of psoriasis and psoriatic arthritis [2]. Secukinumab binds and neutralizes interleukin-17A, preventing it from interacting with IL-17 receptors expressed on keratinocytes, fibroblast-like synoviocytes, endothelial cells, chondrocytes, and osteoblasts [3]. Meanwhile, secukinumab has been shown to block downstream inflammatory pathways that are associated with autoimmune disease. Hemolytic anemia after secukinumab administration was reported first in 2020 by Rivas *et al*. [4]. This is a rare case, but American International Health Alliance (AIHA) is considered to be produced by a number of processes, including antibodies that are either drug-dependent or drug-independent [5]. There is currently no guideline for the treatment of AIHA caused by secukinumab. Here, we reported our patient with autoimmune hemolytic anemia induced secukinumab.

## Patient and observation

**Patient information:** a thirty-nine years old man with an established diagnosis of severe psoriasis vulgaris and psoriatic arthritis accompanied by type 1 diabetes mellitus had finished induction of secukinumab and received the first maintenance dose with the improvement of skin lesions. He was hospitalized for drainage of soft tissue abscess at occipital region.

**Clinical findings:** no remarkable findings besides soft tissue abscess, psoriatic lesion has diminished.

**Diagnostic assessment:** AIHA was confirmed by Direct Antiglobulin Test (DAT) results in +4, antinuclear antibody performed using immunofluorescence with titer 1: 100 and fine speckled pattern. Hemolysis marker, reticulocyte, and LDH were increased and subsequently improved as the hemoglobin levels increased.

**Diagnosis:** diagnostic of AIHA was confirmed by direct antiglobulin test (DAT) with two suspected etiomechanism in this case, which are: drug-induced (secukinumab) and infection (soft tissue abscess). Unfortunately, tissue culture of the infectious specimen was not obtained, and we are unable to perform anti-drug antibody testing and neutralization. We perform Naranjo´s score showing +1 (possible) results for adverse drug events, the findings include previous report for similar adverse reaction (+1), occurrence of adverse events following suspected drug (+2), present of alternative cause (-1), no worsening of symptoms after readmission (-1).

**Therapeutic interventions:** despite clinical improvement after suspected drug rechallenge and no specific treatment regarding AIHA. The Naranjo´s score still shows possibility.

**Follow-up and outcome of interventions:** hemoglobin levels increase, reticulocyte count and LDH decrease, showing improvement in AIHA as shown in Table 1.

**Table 1:** timeline of injection and laboratory changes

Parameters	Date of Treatment (2020)													
	29-04	01-05	08-05	15-05	22-05	29-05	26-06	27-06	24-07	01-08	04-08	07-08	10-08	05-09
Hemoglobin (13 - 17g/dl)	13.5	**Secukinumab Inititation**					12.1	**Secukinumab maintenance**	7.9	9.5	**Secukinumab readministered**	11.5	11.4	15
PCV (39 - 50%)	40.6						37.2		24.1	29.9		36	35.1	46.6
Reticulocyte	-						-		-	9.58		6.44	5.58	1.16
LDH (< 250 U/L)	-						-		-	-		436	420	-
DAT	-						-		-	+4		-	-	-
ANA	-						-		-	-		1:100 fine granular	-	-
C3 (90 - 180 mg/dl)	-						-		-	-		50.3	-	-
C4 (10 - 40 mg/dl)	-						-		-	-		9.41	-	-

**Patient perspective:** patient expecting the continuation of the suspected drug, regarding previous clinical improvement after treatment induction. Consent was obtained, and a monitoring strategy was agreed by patient and treating physician.

**Informed consent:** he had given the authors permission to publish this case report.

## Discussion

Autoimmune hemolytic anemia is common conditions found in clinical practice, despite the elaboration and diagnosis of the etiology remain a challenge for clinicians. Since, treatment will be more efficacious when the underlying etiology has been identified. Classically, AIHA has been classified into: (a) warm type AIHA; (b) cold type AIHA; and (c) mixed type AIHA, by using this classification, the identification of etiology could be assisted. Each type of AIHA has several common etiologies (Table 2) [6]. Hemolysis in AIHA is mediated by an autoantibody to red blood cells, which identified using Direct Antiglobulin Test (DAT) or Coombs´ test, which identify semi-quantitatively the agglutination formation after adding the patient´s blood (which contain RBC coated by autoantibodies) with Coombs´ reagent which contain anti-human antibody resulting of hemagglutination. Despite the conflicting role of DAT in establishing autoimmune hemolytic anemia diagnosis, this method remains widely used. In drug-induced AIHA, Coombs´ test can be positive or negative depending on the aetiomechanism of drug-induced, whether it is immune-mediated or nonimmune-mediated [7]. In our case reported above, despite the hemolytic anemia do not reappear on readministration, Naranjo´s score reveals possible adverse drug reactions as shown in Table 3. In addition, the hemolytic anemia was detected after induction dose, suggesting a dose-effect relationship or concomitant infection after induction.

**Table 2:** classification of autoimmune hemolytic anemia

**Warm AIHA** Primary Secondary Neoplasia (CLL, lymphoma, solid organ) Infection (e.g. hepatitis C, HIV, CMV, VZV, pneumococcal infection, leishmaniasis, tuberculosis)	Immune dysregulation Connective tissue disorders (e.g. SLE, Sjögren syndrome, scleroderma) Ulcerative colitis, PBC, sarcoidosis Post transplantation Immune deficiency syndrome (e.g. CVID)
**Cold AIHA** Cold haemagglutinin disease Primary Secondary Malignancy (e.g. CLL, NHL, solid organ) Infection (e.g. mycoplasma, viral infections including IM)	Autoimmune disease Post-allogeneic HSCT Paroxysmal cold haemoglobinuria Primary Secondary Infection (e.g. adenovirus, influenza A, syphilis, CMV, IM, VZV, measles, mumps, Mycoplasma pneumoniae, Haemophilus influenzae, Escherichia coli)
**Mixed type AIHA** Primary Secondary Lymphoma, SLE, infection	

Note: AIHA, autoimmune hemolytic anemia; CLL, chronic lymphocytic leukaemia; CMV, cytomegalovirus; CVID, common variable immunodeficiency; HIV, human immunodeficiency virus; HSCT, haematopoietic stem cell transplantation; IM, infectious mononucleosis; NHL, non-Hodgkin lymphoma; PBC, primary biliary cirrhosis; SLE, systemic lupus erythematosus

**Table 3:** Naranjo´s score in this case

Question	Yes	No	Unknown
Are there previous conclusive report on this reaction?	**+1**	0	0
Did the adverse event appear after the suspected drug was administered?	**+2**	-1	0
Did the adverse event improve when the drug was discontinued or a specific antagonist was administered?	+1	0	**0**
Did the adverse event reappear when the drug readministered?	+2	**-1**	0
Are there alternative causes that could on their own have caused the reactions?	**-1**	+2	0
Did the reaction reappear when placebo was given?	-1	+1	**0**
Was the drug detected in blood or in other fluids in concentrations known to be toxic?	+1	0	**0**
Was the reaction more severe when the dose was increased or less severe when the dose was decreased?	+1	0	**0**
Did the patient have a similar reaction to the same or similar drugs in any previous exposure?	+1	0	**0**
Was the adverse event confirmed by any objective evidence?	+1	0	**0**
**TOTAL SCORE**	**+1 (Possible)**		

We report our patients that experienced AIHA during treatment of secukinumab, an anti-IL-17A monoclonal antibody used in psoriatic and psoriatic arthritis. IL-17 does play a role in hematopoiesis, despite the exact role and mechanism have not been well identified and also varied. The variation of response leads to the hypothesis that IL-17 action depends on the microenvironment. Several experiments show that IL-17 mostly involves the granulocyte lineage, in healthy mouse bone marrow, IL-17 stimulates myeloid progenitors (Colony-Forming Unit-Granulocytic/Monocytic, CFU-GM) and early-stage erythroid progenitors (Burst-Forming Unit-Erythroid, BFU-E) but inhibits late-stage erythroid progenitors (Colony-Forming Unit-Erythroid, CFU-E) [8]. Hwang, *et al*. studied expression of the surface molecule of Early Erythroid Progenitor (EEP) and Committed Erythroid Progenitor (CEP); he found at 2 ng/ml, IL-17 had a synergistic effect (EC_50_) on erythropoietin. The study shows that addition of 10 ng/ml IL-17 to 0.05 U/ml, increases 2.5-fold CFU-e colonies. IL-17a activated intracellular signaling of STAT3 and STAT5 in EEP [9]. Krstic *et al*. in 2012 suggest the involvement of GATA-1, JNK, and ERK MAPKs in IL-17-regulated erythropoiesis in mice [10]. Hall *et al*. in 2012 studied in 33 subjects with warm type-AIHA and 20 healthy controls, found that IL-17A found to be increased in AIHA subjects compared to control group and correlated with severity of anemia [11].

**Figure 1:**
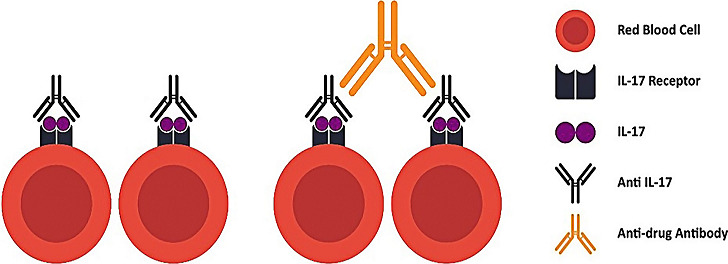
possible interaction between anti IL-17 and red blood cells; mature RBC possibly still express IL-17 receptor and binding of IL-17 with antibody to-IL-17 may results in hemolytic response through several mechanism (i.e complement) or probably agglutination; development of anti-drug antibody may possibly initiate agglutination (illustration: Rosandy KO)

Drug-induced AIHA (DIIHA) is another challenge, Garatty *et al*. (2010) and Garte *et al*. (2011) have reported several drugs known to induced AIHA, several antibiotics, NSAID, and antineoplastic were the most common reported drugs to induce AIHA [6]. To date, monoclonal antibodies rarely reported as the possible etiology of DIIHA, but as biotherapeutic agents, monoclonal antibodies may induce immune-mediated hemolytic anemia through several mechanisms which is autoimmunity, biological effects of drugs, development of anti-drug antibodies, or immune system response to biologic molecule [12]. Development of antibody to anti-IL-17 possibly results in agglutination of RBC if the RBC express IL-17 receptor that bind by anti-IL-17 (Figure 1). Studies show erythroid progenitors express IL-17 receptor, but no data regarding expression of IL-17 receptor in mature RBC, binding of IL-17 to erythroid progenitor stimulate erythropoiesis.

In our patient, ANA detected 1: 1000 and have positive DAT (polyspecific reagent). Unfortunately, we were unable to perform anti-drug antibody testing due to limited resources. Despite the improvement of hemoglobin with continuing secukinumab treatment, Naranjo´s score shows possible results in such adverse drug events. We suggest in such settings, when continuing secukinumab been decided, blood count should be tightly monitored. Furthermore, secukinumab may have been a responsibility for the positive DAT result in our patient, but the infection that occurred in the patient was also a confounding factor. Additional research is needed to investigate the factors that may contribute to secukinumab administration causing AIHA both from internal and external factors.

## Conclusion

We report a patient diagnosed with AIHA after completing an induction dose of secukinumab, but at the same time the patient had a soft tissue infection that can be the precipitating factor for AIHA. IL-17 receptor is expressed by erythroid progenitors, but currently, no data shows the expression of IL-17 receptor in mature red blood cells. On the other side, IL-17A was found to be increased in serum of patients with AIHA showing the role of this cytokine, but no data regarding treatment with anti-IL-17 to suppress immune-mediated hemolysis, but targeting IL-17 could be a future direction. These two conflicting theories need to be elaborate for further study. The possible etiology of AIHA in this patient are: (1) infection-induced hemolysis; (2) drug-induced hemolysis; or (3) concomitant AIHA with psoriatic arthritis and AIHA also respond to anti-IL-17. Finally, additional research is needed to determine the possible mechanism secukinumab-induced AIHA.
